# Overview of point-of-care abdominal ultrasound in emergency and critical care

**DOI:** 10.1186/s40560-016-0175-y

**Published:** 2016-08-15

**Authors:** Toru Kameda, Nobuyuki Taniguchi

**Affiliations:** 1 Department of Emergency Medicine, Red Cross Society Azumino Hospital, 5685 Toyoshina, Azumino, Nagano 399-8292 Japan; 2 Department of Clinical Laboratory Medicine, Jichi Medical University, 3311-1, Yakushiji, Shimotsuke, Tochigi 329-0498 Japan

**Keywords:** Point-of-care ultrasound, Abdominal ultrasound, Emergency, Critical care, Review

## Abstract

Point-of-care abdominal ultrasound (US), which is performed by clinicians at bedside, is increasingly being used to evaluate clinical manifestations, to facilitate accurate diagnoses, and to assist procedures in emergency and critical care. Methods for the assessment of acute abdominal pain with point-of-care US must be developed according to accumulated evidence in each abdominal region. To detect hemoperitoneum, the methodology of a focused assessment with sonography for a trauma examination may also be an option in non-trauma patients. For the assessment of systemic hypoperfusion and renal dysfunction, point-of-care renal Doppler US may be an option. Utilization of point-of-care US is also considered in order to detect abdominal and pelvic lesions. It is particularly useful for the detection of gallstones and the diagnosis of acute cholecystitis. Point-of-case US is justified as the initial imaging modality for the diagnosis of ureterolithiasis and the assessment of pyelonephritis. It can be used with great accuracy to detect the presence of abdominal aortic aneurysm in symptomatic patients. It may also be useful for the diagnoses of digestive tract diseases such as appendicitis, small bowel obstruction, and gastrointestinal perforation. Additionally, point-of-care US can be a modality for assisting procedures. Paracentesis under US guidance has been shown to improve patient care. US appears to be a potential modality to verify the placement of the gastric tube. The estimation of the amount of urine with bladder US can lead to an increased success rate in small children. US-guided catheterization with transrectal pressure appears to be useful in some male patients in whom standard urethral catheterization is difficult. Although a greater accumulation of evidences is needed in some fields, point-of-care abdominal US is a promising modality to improve patient care in emergency and critical care settings.

## Background

Due to the portability and accessibility of ultrasound (US), point-of-care US, which is performed by clinicians at the bedside, is increasingly being used to facilitate accurate diagnoses, to monitor the fluid status, and to guide procedures in emergency and critical care [1]. The main applications in abdominal regions include trauma, biliary, urinary tract, intrauterine pregnancy, and abdominal aortic aneurysm (AAA), which can be evaluated by a transabdominal approach [2, 3]. Additionally, new applications with point-of-care abdominal US have also been assessed and recently proposed. This article provides an up-to-date overview of point-of-care abdominal US performed by clinicians in emergency and critical care settings.

## Review

### Clinical manifestations and point-of-care US

#### Acute abdominal pain

As a single imaging strategy, computed tomography (CT) is overall superior to US in patients with acute abdominal pain [4]. Laméris et al. reported that conditional strategy with CT after negative or inconclusive radiology US resulted in the highest overall sensitivity, with only 6 % missed urgent conditions, and the lowest overall exposure to radiation by performing CT in only half of adult patients with acute abdominal pain [4]. In this regard, imaging strategies including point-of-care abdominal US must also be evaluated.

A pilot observational study showed that emergency physician (EP)-performed US appears to positively impact decision-making and the diagnostic workup of patients with nonspecific abdominal pain as determined by the nursing triage. In 128 patients, 58 (45 %; 95 % confidence interval (CI), 36–54 %) had an improvement in diagnostic accuracy and planned diagnostic workup using US [5]. In a randomized study including 800 adult patients with acute abdominal pain, Lindelius et al. reported the utility of US performed by surgeons who underwent a 4-week US training program. The proportion of correct primary diagnoses was 7.9 % higher in the group undergoing surgeon-performed US than in the control group (64.7 vs 56.8 %; *p* = 0.027) [6]. The number of US performed in the radiology department was significantly lower in the group receiving surgeon-performed US, while there was no difference between the groups regarding the number of ordered CT scans or other examinations [7].

Evidence on detection of each lesion causing acute abdominal pain with point-of-care abdominal US is reviewed in the “Detection of abdominal and pelvic lesions” section. Methods for the assessment of acute abdominal pain with point-of-care US must be developed according to the accumulated evidence in each abdominal region.

#### Hemoperitoneum

Abdominal US in trauma patients is typically performed with the methodology of a focused assessment with sonography for trauma (FAST) examination. FAST provides a quick overview of the peritoneal cavity to detect free fluid, which is a direct sign of hemoperitoneum and an indirect sign of organ injuries. The sensitivity and specificity of FAST for the detection of free intraperitoneal fluid were 64–98 and 86–100 %, respectively. These ranging results may be explained by differences in the levels of clinical experience and in the reference standards [8]. The sensitivity may be higher, and time needed to perform may be shorter in patients with hemodynamic collapse. Wherrett et al. demonstrated that an abdominal assessment with FAST required 19 ± 5 s in the positive group and 154 ± 13 s in the negative group (*p* < 0.001) with high accuracy in 69 hypotensive blunt trauma patients [9].

It is also reasonable to consider the usage of a complete or partial FAST examination in evaluating spontaneous hemoperitoneum in non-trauma patients. The etiology of spontaneous hemoperitoneum can vary, and the causes may be classified as gynecologic, hepatic, splenic, vascular, or coagulopathic conditions [10]. Spontaneous hemoperitoneum frequently presents with acute abdominal pain with or without hemodynamic collapse. In some patients, the collapse becomes obvious after the initial evaluation; therefore, spontaneous hemoperitoneum should be detected rapidly during the evaluation. Case reports comment on the use of bedside US to detect intra-abdominal free fluid to aid in the diagnosis of the causes; however, few original studies have explored its use [11].

Hemoperitoneum caused by gynecologic conditions, such as rupture of the gestational sac in ectopic pregnancy and hemorrhage or rupture of an ovarian cyst, is common in women of childbearing age, in whom US is selected as the primary imaging modality [10]. In a retrospective study, Rodgerson et al. demonstrated that identifying patients with a suspected ectopic pregnancy and fluid in Morison’s pouch by EP-performed abdominal US decreased the time to diagnosis and treatment [12]. In a prospective observational study, Moore et al. reported that ten of 242 patients with suspected ectopic pregnancy were found to have fluid in Morison’s pouch with EP-performed abdominal US, and nine of the ten patients underwent immediate operative intervention for ruptured ectopic pregnancy. They concluded that free intraperitoneal fluid in Morison’s pouch in patients with suspected ectopic pregnancy may be rapidly identified by US and predicts the need for intervention [13].

**Fig. 1:**
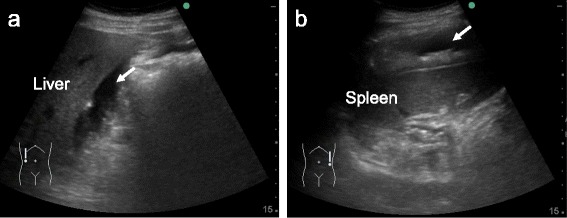
Ultrasound images in a 47-year-old man who presented with left upper continuous abdominal pain. The patient began to feel pain after heavy physical labor without awareness of a traumatic event. Bedside ultrasound after history taking and a physical examination revealed free fluid in Morison’s pouch (**a**, *arrow*), perisplenic space (**b**, *arrow*), and rectovesical pouch. Contrast-enhanced computed tomography showed hemoperitoneum and splenomegaly with a low-density, striped area in the lower pole. He was diagnosed as having splenic rupture and treated conservatively

However, US is not sensitive at identifying a focus of extravasation from a vessel or organ [8]. Therefore, FAST may be an option for the initial evaluation to detect hemoperitoneum in non-trauma patients (Fig. 1).

#### Hypoperfusion and renal dysfunction

Doppler US is indicated as a tool to assess renal perfusion. The Doppler-based resistive index (RI) is calculated using the following formula: (peak systolic velocity − end-diastolic velocity)/peak systolic velocity in an interlobar or arcuate artery, with a normal value of 0.58 ± 0.10. It is broadly accepted that values >0.70 are considered to be abnormal [14]. Corradi et al. reported that in normotensive polytrauma patients without biochemical signs of hypoperfusion, a renal Doppler RI greater than 0.7 at admittance into the emergency department was predictive of hemorrhagic shock within the first 24 h (odds ratio, 57.8; 95 % CI, 10.5–317.0; *p* < 0.001). However, the inferior vena cava (IVC) diameter and caval index were not predictive in these patients. They hypothesized that most of the patients were normovolemic at arrival [15]. Although larger comparative studies are needed, a high renal Doppler RI may be more predictive of hemorrhagic shock than the IVC diameter and caval index [15].

A renal Doppler RI may also help in detecting early renal dysfunction or predicting short-term reversibility of acute kidney injury (AKI) in critically ill patients [16–18]. A preliminary study showed that a semi-quantitative assessment of renal perfusion using color Doppler was easier to perform than the RI and may provide similar information [16]. That study also found that both the semi-quantitative assessment using color Doppler and the RI could be performed with good feasibility and reliability by inexperienced operators, such as intensive care residents following a half-day training session [16]. Doppler US may be useful in assessing renal perfusion; however, larger studies with standardized methods are needed to confirm these results and reveal its roles in the management of patients with AKI [19].

### Detection of abdominal and pelvic lesions

#### Gallstone and acute cholecystitis

It is well known that radiology US is very useful for the detection of gallstones and the diagnosis of acute cholecystitis [20]. A systematic review and meta-analysis was conducted to compare surgeon-performed US for suspected gallstone disease to radiology US or a pathological examination as the gold standard investigation. The search criteria resulted in eight studies with 1019 patients. The pooled sensitivity was 96 % (95 % CI, 93.4–97.9 %), and the specificity was 99 % (95 % CI, 98.3–99.8 %) [21]. On the other hand, EP interpretation for the identification of gallstones is reported to have a sensitivity of 86–96 % and specificity of 78–98 % [22].

Gallstones are found in approximately 95 % of patients with acute cholecystitis; however, the detection of gallstones is not specific for the diagnosis of acute cholecystitis. When performing US, secondary findings such as gallbladder wall thickening, pericholecystic fluid, and sonographic Murphy sign provide more specific information [20]. Summers et al. reported in a prospective observational study with 164 enrolled patients that the test characteristics of EP-performed US for the detection of acute cholecystitis had a sensitivity of 87 % (95 % CI, 66–97 %), specificity of 82 % (95 % CI, 74–88 %), positive predictive value of 44 % (95 % CI, 29–59 %), and negative predictive value of 97 % (95 % CI, 93–99 %). Additionally, the test characteristics of EP-performed US were similar to those of radiology US. According to the high negative predictive value, the study indicated that patients with a negative result are unlikely to require cholecystectomy or admission within 2 weeks of their initial presentation [23].

#### Appendicitis

CT was found to have a superior test performance to US in the diagnosis of acute appendicitis; however, US is recommended as the first-line imaging modality in young, female, and slender patients in view of the radiation exposure [24]. Recent studies from the field of emergency medicine addressed the diagnostic performance of point-of-care US performed by EPs or pediatric EPs in the evaluation of suspected appendicitis [25–30] (Table 1). In these studies, no visualization of the appendix with US was coded as a negative result, and the final diagnosis of appendicitis was made with operative or pathology findings. Chen et al. demonstrated a high sensitivity in their study, where more extensive US training was provided and the prevalence of appendicitis was higher [25]. Several studies demonstrated the feasibility of reducing the length of stay in the emergency department [28] and avoiding CT according to the result of a high positive predictive value in some patients [30] when using point-of-care US as the first-line imaging modality. To date, the diagnosis of appendicitis with point-of-care US by clinicians has not been fully accepted. A large prospective study is necessary to investigate methods to increase the accuracy of point-of-care US through more effective educational techniques and safety of the addition to sequential radiology imaging [28, 30].

**Table 1:** Diagnostic performance of ultrasound performed by emergency physicians in the evaluation of suspected acute appendicitis

Author	Sample size	Prevalence (%)	Sensitivity (%)	Specificity (%)	PPV (%)	NPV (%)
Chen et al. [25]	147	75	96	68	90	86
Fox et al. [26]	155	45	39	90	75	65
Fox et al. [27]	126	45	65	90	84	76
Elikashvili et al. [28]	150	33	60	94	86	82
Sivitz et al. [29]	264	32	85	93	85	93
Mallin et al. [30]	97	35	68	98	96	85

Four studies [25, 27–30] were performed prospectively. The final diagnosis of appendicitis was made according to operative or pathology findings

*PPV* positive predictive value, *NPV* negative predictive value

#### Small bowel obstruction

The utility of surgeon-performed US for the diagnosis of bowel obstruction and early recognition of strangulation was evaluated in the 1990s [31]. In recent years, some studies showed the accuracy of EP-performed US for the diagnosis of small bowel obstruction. Unlüer et al. demonstrated in a prospective study with 168 patients that the sensitivity and specificity were 97.7 % (95 % CI, 94.5–100 %) and 92.7 % (95 % CI, 87.0–98.3 %), respectively. Additionally, the diagnostic accuracy of EP-performed and radiology-performed US were not statistically different from one another [32]. Jang et al. demonstrated in a prospective study with 76 patients that the sensitivity and specificity were 90.9 % (95 % CI, 74.5–97.6 %) and 83.7 % (95 % CI, 68.7–92.7 %), respectively [33]. These studies also showed that EP-performed US had a superior test performance compared with an X-ray in the diagnosis of small bowel obstruction [32, 33]. However, large prospective studies are needed to alter the management of small bowel obstruction with its use.

#### Gastrointestinal perforation

**Fig. 2:**
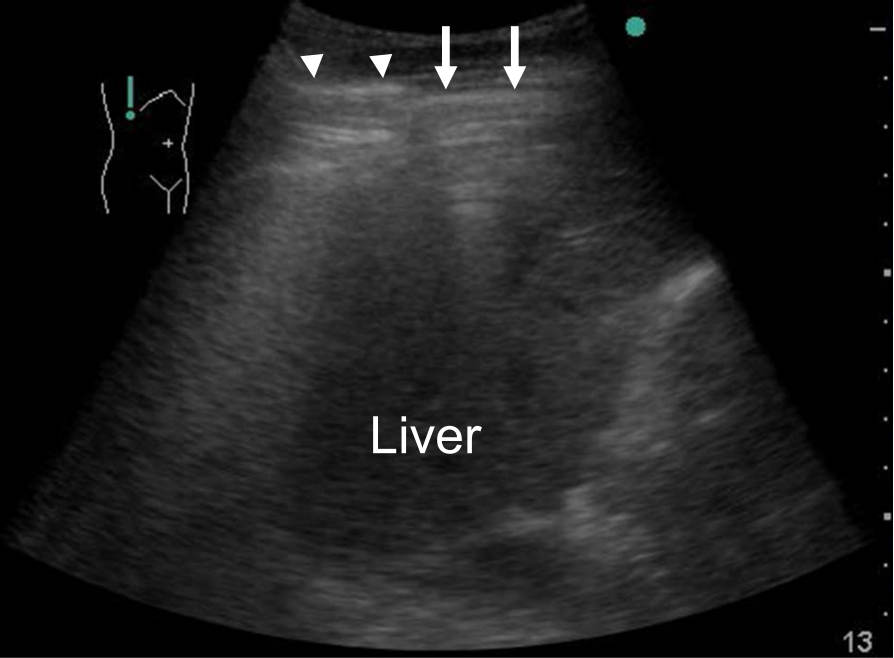
An ultrasound image in a 43-year-old man who presented with sudden onset of abdominal pain. The patient had a history of a duodenal ulcer and was aware of black stool prior to the presentation. On physical examination, he had diffuse abdominal tenderness with guarding. Bedside ultrasound was performed with the patient in the left lateral decubitus position. Reverberation artifacts on the ventral surface of the liver (*arrows*) indicated intraperitoneal free air. The artifacts were distinguished from other artifacts with respiratory movement (*arrowheads*), which originated at the lung surface

The diagnosis of gastrointestinal perforation is based on the evidence of pneumoperitoneum, which is usually detected with an X-ray or CT. A US sign of pneumoperitoneum (Fig. 2) has also been recognized following a comprehensive study on visualizing pneumoperitoneum with US reported from Germany over 30 years ago [34]. In the 21st century, the utility of clinician-performed US for the detection of pneumoperitoneum was reported from Asian countries. Prospective studies have demonstrated the sensitivity and specificity to be 85–93 % and 53–100 %, respectively [35–37]. Moreover, Chan et al. also reported that US was more sensitive than an X-ray for the detection [36]. However, large prospective trials are needed to validate the accuracy of this modality and whether the concept can be generalized among clinician sonographers.

#### Ureterolithiasis and pyelonephritis

Pain due to ureterolithiasis is a common problem in the emergency room. CT has become the most common initial imaging modality for suspected ureterolithiasis because of its high accuracy [38]. However, CT exposes patients to ionizing radiation, which is especially concerning for patients with ureterolithiasis as they are prone to recurrence and repeated imaging. Moreover, no evidence has shown that increased CT use is associated with an improved patient outcome [38]. The diagnostic performance of bedside US performed by EPs or medical staff members in the diagnosis of ureterolithiasis has been prospectively studied, as shown in Table 2 [39–42]. These studies, which used CT as the reference standard, showed that the diagnostic performance using US finding of hydronephrosis was generally modest. In one of the articles, Herbst et al. also demonstrated that attending physicians with fellowship training had significantly better sensitivity than all other users (93 vs 68 %) [42]. A large, multicenter, randomized trial conducted in the USA showed that initial US performed by EPs was associated with lower cumulative radiation exposure than initial CT, without significant differences in high-risk diagnoses with complications, serious adverse events, pain scores, return emergency department visits, or hospitalizations [38]. Although US was less sensitive than CT for the diagnosis of ureterolithiasis, bedside US in emergency departments is justified as the initial imaging modality. Moreover, whether the detection of the stone itself in addition to hydronephrosis with point-of-care US actually improves the accuracy of the diagnosis requires further investigation [43].

**Table 2:** Diagnostic performance of ultrasound performed by emergency clinicians in the evaluation of suspected ureterolithiasis

Author	Sample size	Prevalence (%)	Sensitivity (%)	Specificity (%)	PPV (%)	NPV (%)
Gaspari et al. [39]	104	51	87	82	84	86
Watkins et al. [40]	57	68	80	83	91	65
Moak et al. [41]	107	36	76	78	66	86
Herbst et al. [42]	670	47	73	73	71	75

*PPV* positive predictive value, *NPV* negative predictive value

**Fig. 3:**
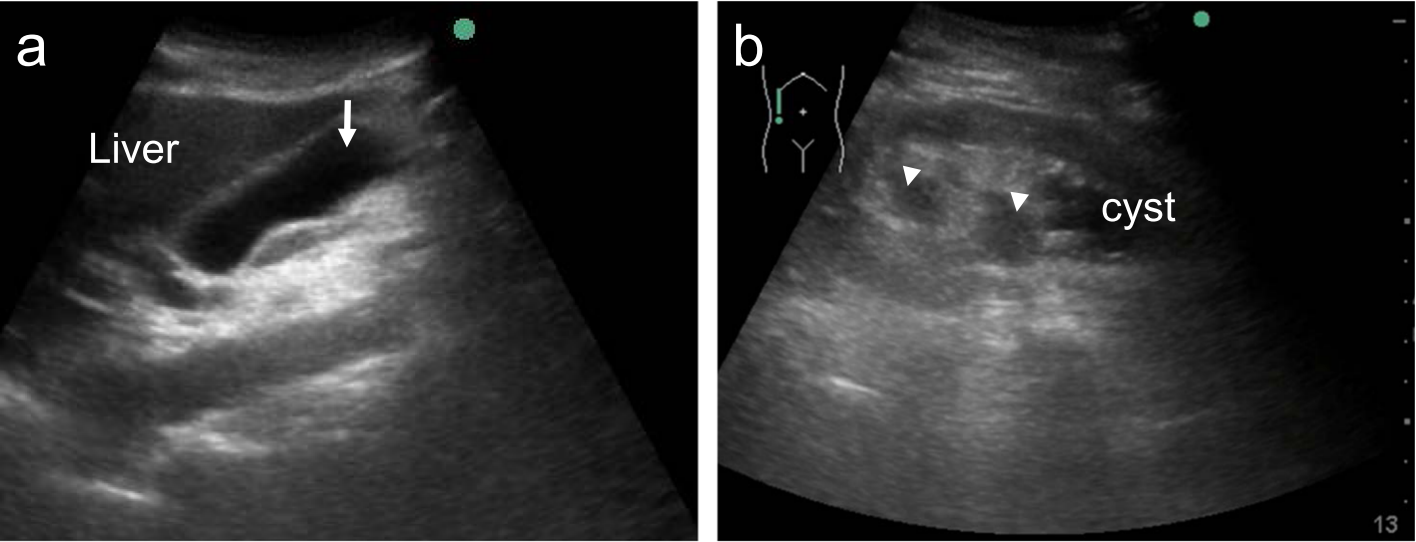
Ultrasound images in an 88-year-old man who presented with shaking chills. The patient had a history of acute cholecystitis with percutaneous transhepatic gallbladder drainage. On physical examination, he had no abdominal or costovertebral angel tenderness. Bedside ultrasound showed a normal gallbladder (**a**, *arrow*) and pelvic dilatation in the right kidney (**b**, *arrowheads*). A subsequent computed tomography scan revealed the stone at the right ureterovesical junction. A complicated urinary tract infection was strongly suspected, and emergent urological consultation was ordered. He fell into shock soon after the initial evaluation

Acute pyelonephritis is also a common disease encountered in emergency departments. For complicated acute pyelonephritis, such as obstructive uropathy due to ureterolithiasis, delayed management can lead to high morbidity and mortality. Chen et al. showed that EP-performed US was able to detect significant abnormalities such as hydronephrosis, polycystic kidney disease, renal abscess, and emphysematous pyelonephritis in 40 % of patients finally diagnosed with acute pyelonephritis. The early utilization of US in emergency departments may impact on the management of these patients or initial assessment of septic patients [44] (Fig. 3).

#### Adnexa and uterus

It has been generally accepted that transvaginal US is superior to transabdominal US for evaluating adnexa and uterus, and transvaginal US is generally selected as the initial technique among gynecological imaging modalities [45]. In some institutions and countries, EPs perform transvaginal US in daily practice; however, EP-performed transvaginal US is not common globally. They may have the opportunity to perform transabdominal US in women who may have genital problems [45].

A systematic review and meta-analysis showed that the use of bedside transabdominal US and/or transvaginal US performed by EPs consistently exhibits excellent test characteristics for ruling out ectopic pregnancy. In this investigation, the positive and negative results were defined as the absence of a definite intrauterine pregnancy and a visible intrauterine pregnancy, respectively. Ten studies were included with a total of 2057 patients, of whom 152 (7.5 %) had an ectopic pregnancy. The pooled sensitivity and negative predictive value were reported to be 99.3 % (95 % CI, 96.6 to 100 %) and 99.96 % (95 % CI, 99.6 to 100 %), respectively [46].

As mentioned previously, point-of-care transabdominal US is useful to detect hemoperitoneum due to gynecologic diseases. Moreover, it is reasonable to investigate its efficacy to detect genital lesions themselves, because the use of point-of-care transabdominal US as an extension of the physical examination is rapidly growing with widespread application [45].

#### AAA

The use of US performed by EPs to diagnose AAA has been well studied prospectively since the 2000s. A systematic review and meta-analysis published in 2013 showed that the search criteria resulted in seven studies with 655 patients, and the pooled operating characteristics of EP-performed US for the detection of AAA had a sensitivity of 99 % (95 % CI, 96–100 %) and specificity of 98 % (95 % CI, 97–99 %) [47]. Bedside US can be used with great accuracy to detect AAA in symptomatic patients; therefore, it is justified as the initial imaging modality to rapidly detect AAA in emergency departments.

### Usages assisting procedures

#### Paracentesis

US guidance enables visualization of the needle insertion site to perform paracentesis safely. An observational cohort study using a nationally representative database was conducted to examine the effect of US guidance on the risk of bleeding complications after paracentesis. Of 69,859 patients undergoing paracentesis, 0.8 % (*n* = 565) experienced bleeding complications. After adjusting for the inpatient or outpatient procedures, the duration of hospitalization before the paracentesis, and the admission diagnoses, US guidance reduced the risk of bleeding complications by 68 % (odds ratio, 0.32; 95 % CI, 0.25–0.41). The data indicated that US guidance is associated with a decreased risk of complications after paracentesis [48]. A randomized study with 100 enrolled patients demonstrated that the success rate of US-assisted paracentesis performed by EPs with varying levels of experience and the traditional technique were 95 and 65 %, respectively (*p* = 0.0003) [49]. Case series indicated that emergent US-guided paracentesis may lead to a significant management change in selected unstable patients with a positive FAST examination [50]. As mentioned above, paracentesis under US guidance is shown to improve patient care. Furthermore, localization of the inferior epigastric artery before paracentesis may provide a more reliable means to avoid complications [51].

#### Conformation of gastric tube placement

Gastric tube insertion is commonly performed in emergency and critical care settings. Immediately after the procedure, the placement of the tube is typically evaluated using a visual inspection of aspirate contents and auscultation with instillation of air in the tube. Additionally, a chest X-ray is recommended in most cases to confirm correct placement. However, a chest X-ray has issues, including radiation exposure, delayed confirmation, and cost. Several recent studies showed that US is a potential modality to verify the placement of the gastric tube. The methods include confirmation of the tube in the stomach [52], the stomach or duodenum with or without instillation of normal saline mixed with air [53], and the cervical esophagus and stomach with or without instillation of air [54] or normal saline with air [55]. The visualization can be affected by the size of the tube [52] and volume of gas in the gastrointestinal tract [55]. If the presence of the tip of the tube in the stomach is verified with direct visualization or an indirect finding of dynamic fogging made by the instillation, US in addition to physical examinations appears to be a substitute imaging modality for a chest X-ray in some patients.

#### Urethral catheterization

Urethral catheterization is frequently performed for a urinalysis and culture, management of acute urinary retention, and monitoring of the urine output in emergency and critical care settings.

If there is little certainty of the presence or amount of urine in the bladder before urethral catheterization, then this procedure to obtain urine for an analysis and culture often needs to be repeated. The estimation of the amount of urine using bedside bladder US has been reported to lead to an increased success rate during the first attempt in children younger than 2 years of age [56, 57].

**Fig. 4:**
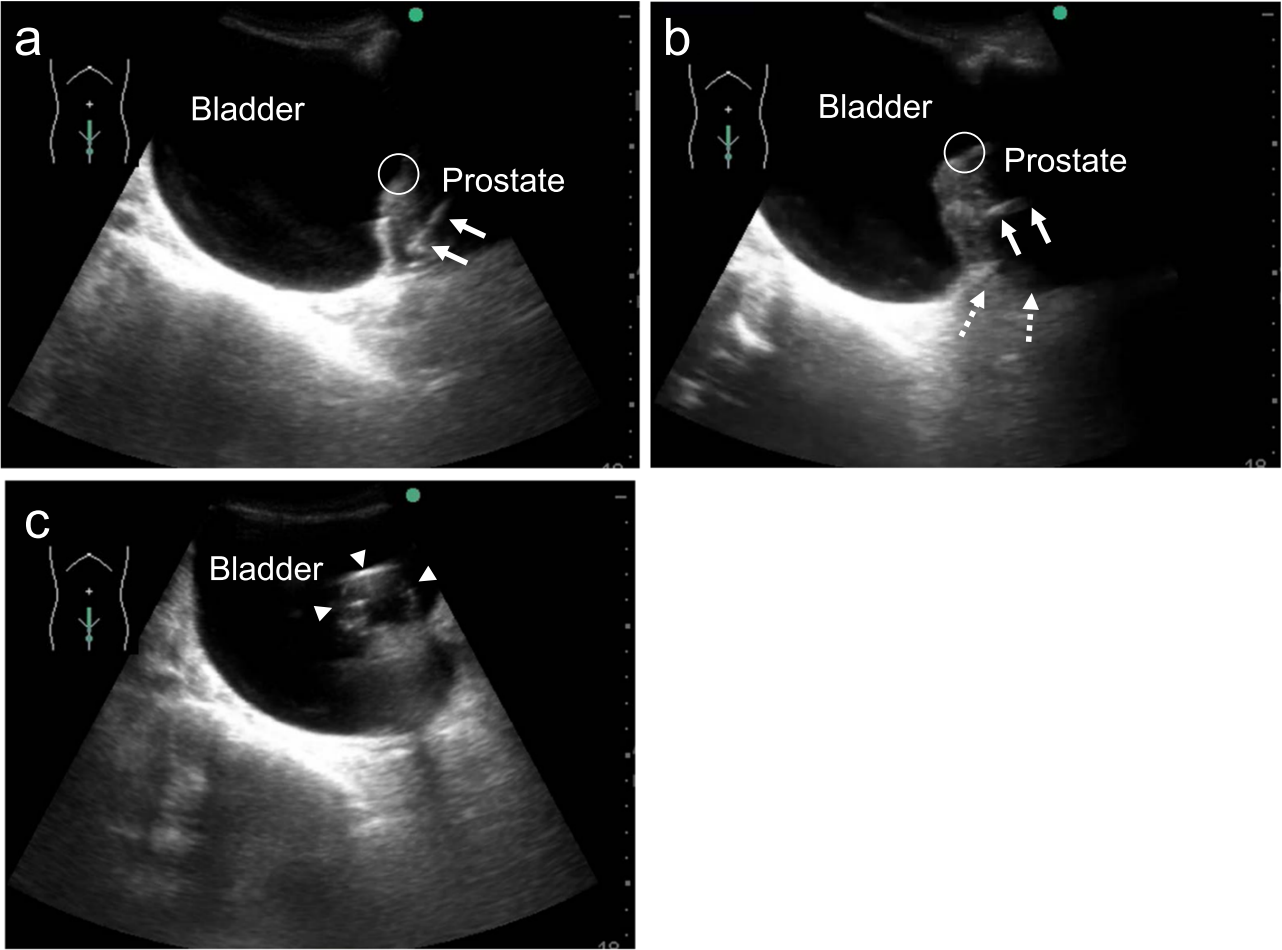
Ultrasound images in a 78-year-old man who presented with difficult urination. The patient had a history of benign prostatic hypertrophy. Standard urethral catheterization attempted by an experienced emergency nurse and an experienced emergency physician failed due to complicated urethral bleeding. **a** Bedside ultrasound revealed the tip of the catheter in a part of the posterior and bulbar urethra (*arrows*) while the progress was obstructed. Judging from the location of the internal urethral orifice, a part of the urethra was thus determined to be bent. The *circle* denotes the location of the internal urethral orifice. **b** The bent part of the urethra had become blunt with transrectal pressure using an inserted index finger (*broken arrows*). The *arrows* denote the tip of the catheter, and the *circle* denotes the location of the internal urethral orifice. **c** Ultrasound-guided catheterization with transrectal pressure without forceful manipulation was successful on the first attempt. *Arrowheads* denote the inflated balloon

In adult male patients, difficulty with standard catheterization is occasionally encountered. In such cases, repeated and unsuccessful blind attempts can cause patient distress and damage to the urethra, usually requiring a urological consultation. Kameda et al. mentioned in their pilot study that transabdominal US performed by emergency medical personnel can reveal the tip of the catheter in a part of the posterior and bulbar urethra, and US-guided catheterization with transrectal pressure appears to be safe and useful in some male patients in whom standard urethral catheterization is difficult [58] (Fig. 4).

## Conclusions

Methods for the assessment of acute abdominal pain with point-of-care abdominal US must be developed according to the accumulated evidence in each abdominal region. To detect hemoperitoneum, a FAST examination may be a helpful option in non-trauma patients. For the assessment of systemic hypoperfusion and renal dysfunction, point-of-care renal Doppler US may be an option. The utilization of point-of-care US is also considered in order to detect abdominal and pelvic lesions. It is useful for the detection of gallstones and the diagnosis of acute cholecystitis. It is justified as the initial imaging modality for the diagnosis of ureterolithiasis and the assessment of pyelonephritis. It can be used with great accuracy to detect the presence of AAA in symptomatic patients. It may also be useful for the diagnoses of digestive tract diseases. Additionally, point-of-care US can be a modality for assisting procedures. Paracentesis under US guidance is shown to improve patient care. US appears to be a potential modality to verify the placement of a gastric tube. Moreover, the estimation of the amount of urine with bladder US can lead to an increased success rate in small children. US-guided catheterization with transrectal pressure appears to be useful in some male patients in whom standard urethral catheterization is difficult. Although a greater accumulation of evidence is needed in some fields, point-of-care abdominal US is a promising modality to improve patient care in emergency and critical care settings.

## Abbreviations

AAA, abdominal aortic aneurysm; AKI, acute kidney injury; CI, confidence interval; CT, computed tomography; EP, emergency physician; FAST, focused assessment with sonography for trauma; IVC, inferior vena cava; RI, resistive index; US, ultrasound
